# Glycosylation of Recombinant Antigenic Proteins from *Mycobacterium tuberculosis*: In Silico Prediction of Protein Epitopes and Ex Vivo Biological Evaluation of New Semi-Synthetic Glycoconjugates

**DOI:** 10.3390/molecules22071081

**Published:** 2017-06-29

**Authors:** Teodora Bavaro, Sara Tengattini, Luciano Piubelli, Francesca Mangione, Roberta Bernardini, Vincenzina Monzillo, Sandra Calarota, Piero Marone, Massimo Amicosante, Loredano Pollegioni, Caterina Temporini, Marco Terreni

**Affiliations:** 1 Department of Drug Sciences, University of Pavia, via Taramelli 12, I-27100 Pavia, Italy; teodora.bavaro@unipv.it (T.B.); sara.tengattini01@ateneopv.it (S.T.); marco.terreni@unipv.it (M.T.); 2 Department of Biotechnology and Life Sciences, University of Insubria, via J.H. Dunant 3, I-21100 Varese, Italy; Luciano.Piubelli@uninsubria.it (L.P.); loredano.pollegioni@uninsubria.it (L.P.); 3 The Protein Factory, Interuniversity Centre Politecnico of Milano and University of Insubria, via Mancinelli 7, I-20131 Milano, Italy; 4 Microbiology and Virology Unit, IRCCS San Matteo Hospital Foundation, viale Camillo Golgi 19, I-27100 Pavia, Italy; francesca.mangione01@universitadipavia.it (F.M.); vincenzina.monzillo@unipv.it (V.M.); s.calarota@smatteo.pv.it (S.C.); pmarone@smatteo.pv.it (P.M.); 5 Department of Biomedicine and Prevention and Animal Technology Station, University of Rome “Tor Vergata”, via Montpellier 1, I-00133 Roma, Italy; Roberta.Bernardini@uniroma2.it (R.B.); amicosan@uniroma2.it (M.A.); 6 Infection Disease Unit, Internal Medicine and Medical Therapy Department, University of Pavia, via Aselli 43/45, I-27100 Pavia, Italy

**Keywords:** *neo*-glycoproteins, glycoconjugate vaccines, MTB recombinant antigens, epitope

## Abstract

Tuberculosis is still one of the most deadly infectious diseases worldwide, and the use of conjugated antigens, obtained by combining antigenic oligosaccharides, such as the lipoarabinomannane (LAM), with antigenic proteins from *Mycobacterium tuberculosis* (MTB), has been proposed as a new strategy for developing efficient vaccines. In this work, we investigated the effect of the chemical glycosylation on two recombinant MTB proteins produced in *E. coli* with an additional seven-amino acid tag (recombinant Ag85B and TB10.4). Different semi-synthetic glycoconjugated derivatives were prepared, starting from mannose and two disaccharide analogs. The glycans were activated at the anomeric position with a thiocyanomethyl group, as required for protein glycosylation by selective reaction with lysines. The glycosylation sites and the ex vivo evaluation of the immunogenic activity of the different *neo-*glycoproteins were investigated. Glycosylation does not modify the immunological activity of the TB10.4 protein. Similarly, Ag85B maintains its B-cell activity after glycosylation while showing a significant reduction in the T-cell response. The results were correlated with the putative B- and T-cell epitopes, predicted using a combination of in silico systems. In the recombinant TB10.4, the unique lysine is not included in any T-cell epitope. Lys30 of Ag85B, identified as the main glycosylation site, proved to be the most important site involved in the formation of T-cell epitopes, reasonably explaining why its glycosylation strongly influenced the T-cell activity. Furthermore, additional lysines included in different epitopes (Lys103, -123 and -282) are also glycosylated. In contrast, B-cell epitopic lysines of Ag85B were found to be poorly glycosylated and, thus, the antibody interaction of Ag85B was only marginally affected after coupling with mono- or disaccharides.

## 1. Introduction

Tuberculosis (TB) remains one of the most relevant public health problems worldwide with a high prevalence, morbidity, and mortality [1]. Like the deadly association with HIV, the diffusion of multidrug resistant (MDR) and extensively drug resistant (XDR) strains of *Mycobacterium tuberculosis* (MTB) is posing additional challenges in TB control [1]. Preventive measures, such as an efficient vaccine, together with diagnostic tools for identifying active TB cases early on are needed [1,2,3]. Both of these aspects rely on the use of highly immunogenic antigens of MTB. At present, however, an efficient, protective anti-TB vaccine capable of replacing the old *M. bovis* Bacillus Calmette-Guérin (BGG) vaccine is still missing [1,2]. In addition to the use of novel and infection phase-specific, protein antigens for the development of anti-TB vaccines and immunodiagnostic tools [3,4], improving immunogenicity by modifying the MTB proteins could play a key role in defining a novel set of bio-tools for TB control.

The glycosylation or other controlled chemical modifications of proteins, such as PEGylation and acylation, can dramatically improve their physical and biological properties [5]. Glycoproteins have been largely investigated for the study of new therapeutic strategies [6], with particular relevance in the development of carbohydrate-based vaccines [7,8]. For instance, *neo*-glycoproteins, which contain novel, designed chemical linkages between protein and saccharides, can provide carbohydrate antigens and immunogens from which immunodiagnostic and therapeutic agents can be derived [9]. In this context, the conjugation of a protein with a moiety of synthetic carbohydrates might strongly increase protein antigenicity, increasing the CD4+ and CD8+ T-cell responses by up to 50-fold as a consequence of improved antigen uptake [10]. This can provide the rationale for designing and developing new vaccine products showing an efficient delivery and uptake of the antigen [7,11] as well as new T cell-based immunodiagnostic tests for TB with an increased sensitivity, owing to a better antigen presentation and T-cell stimulation [12]. Accordingly, conjugation of antigenic proteins from MTB with arabinomannane polysaccharides has been proposed for developing highly immunogenic glycoconjugate vaccines active against TB [7].

Chemical routes for synthesizing *neo-*glycoproteins can involve random or site-selective modifications of protein surface residues, whereby the final covalent linkage of the glycans via their reducing end interposed by a reactive spacer [6,13] is expected to react with nucleophilic side chains of lysine or cysteine residues [8,14,15,16,17]. One example of the first strategy implies the use of 2-iminomethoxyethyl thioglycosides (IME) and takes advantage of the high abundance of lysine residues on the protein surface, allowing various saccharide units for each molecule of protein to be introduced [15,18,19]. However, the coupling reaction between oligosaccharides and the protein by a nonselective glycosylation approach induces formation of different and randomly modified glycoforms, which can result in a decrease/loss of such a biological activity [20]. In the case of antigenic proteins, glycosylation can shield antigenic patches reducing the recognition by the mediator of the immunological response (such as antibodies).

For antigenic proteins with well-defined epitopic sequences, the use of synthetic peptides and glycopeptides has been proposed for developing glycosylated antigens with improved properties, involving the selective glycosylation of residues placed far from the epitope [20]. However, for non-characterized antigenic proteins, this strategy would be difficult to use because a large number of peptides and glycopeptides would need to be prepared as putative antigenic sequences. Alternatively, characterization of epitopes could be performed by mutagenic approach (for example, by alanine scanning).

However, an alternative and suitable approach for the development of efficient glycoconjugate products might also be the preparation of semi-synthetic *neo-*glycoproteins, coupled to a detailed analytical characterization of the products. In this way, information on the involvement of the most reactive lysines in the protein epitopes, and on the effect of their glycosylation on the biological activity might be obtained. For this purpose, recently, a combination of electrospray ionization -mass spectrometry (ESI-MS) and liquid chromatography-mass spectrometry (LC-MS) analytical methods was applied to characterize reactivity in the glycosylation reaction of different residues on the surface of TB10.4, the simplest antigenic protein isolated from MTB [21]. This approach was associated to a computational analysis of the residues involved in the B-cell epitopes formation, and IME activation was proposed for selective glycosylation of the single lysine residue of this protein, avoiding the glycosylation of the putative antigenic sites [21]. On the contrary, for proteins containing multiple lysine residues, the glycosylation process could be designed to emphasize selectivity towards the most reactive lysine residues [22].

A study of the effect of the glycosylation on the antigenic activity, combined to the exact analytical characterization of the glycosylation sites and to computational analysis, has been carried out to define the most relevant epitopic sites of two MTB proteins. Accordingly, we prepared and characterized a number of glycoderivatives obtained by conjugating different glycans (mannose, dimannose, arabinose-mannose), activated with the IME reactive group, with two recombinant MTB protein antigens (rTB10.4 and rAg85B) [23]. In order to assess the effect of the glycosylation on their immunogenicity, site occupancy and protein conformation of the different glycoderivatives were studied. In this study, simple glycans (mono and disaccharides) have been considered in order to minimize the effect of the glycosylaton on the 3D structure of the target proteins.

The biological properties of the glycosylated TB10.4 and Ag85B resulting from ex vivo biological assays were investigated, including ELISPOT assay to evaluate T-cell response and ELISA assay to assess antibody response. The experimental evidence for structure and activity of the glycosylated antigens was correlated with data from an extensive B- and T-cell epitope prediction analysis of the native proteins. The information we obtained was then used to derive structure–activity relationships that could be useful for the rational optimization of the *neo-*glycoconjugate products.

## 2. Results and Discussion

### 2.1. Neo-glycoprotein Preparation and Characterization

**Scheme 1:**
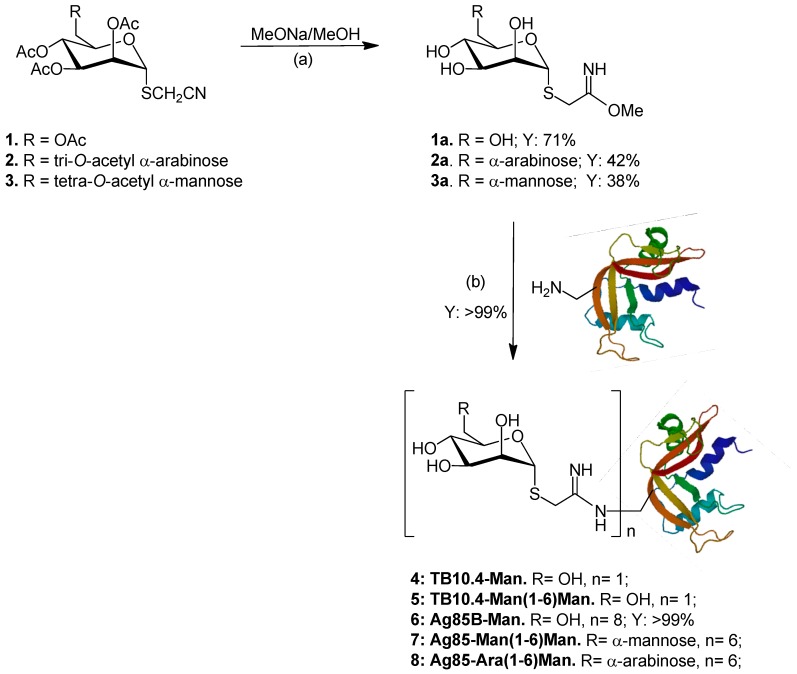
Synthesis of *neo-*glycoproteins **4**–**8** by conjugation reaction of IME saccharides **1a**–**3a** with TB10.4 and Ag85B: (**a**) **1**, **2** and **3**, MeONa/MeOH, room temperature, 24 h, 50% yield; and (**b**) **1a**–**3a**, sodium tetraborate buffer, pH 9.5, 37 °C (25 °C for product **5**), molar ratio glycosidic reagent/(TB10.4/Ag85B) 200:1, 24 h.

The different glycans (**1**–**3**, Scheme 1) bearing a thiocyanomethyl group in the anomeric position were chemo-enzymatically synthesized and activated to obtain intermediates **1a**–**3a** (Scheme 1) according to the procedure previously reported [22]. The activation yields were derived after direct infusion (DI) of the glycosides in ESI-MS.

Following the previously reported procedure [23], both rTB10.4 and rAg85B were produced that contained a seven-amino acid pre-sequence (AMAISDP) at the N-terminal end. Accordingly, rTB10.4 was obtained as a 103-amino acid sequence including only one lysine (Lys100), corresponding to Lys93 in the native protein: the average molecular mass of this recombinant protein is 11,076.3 Da. The rAg85B was obtained as a 292-amino acid protein, which includes eight lysines and possesses an average molecular mass of 31,345.6 Da [23]. Before glycosylation, both proteins were characterized in terms of identity and purity by intact mass measurement (Figure S1A,D). The final protein preparations do not contain endotoxins.

The two recombinant MTB proteins were glycosylated with the different activated sugars [21] and the glycosylation degree of each coupling reaction was monitored by DI-ESI-MS. The deconvoluted spectra (Figure S1B,C,E,F,G) demonstrate that quantitative glycosylation was obtained in all cases. For rTB10.4, the monoglycosylated species was the main product, with a di-glycosylated derivative present in both products 4 (30–40%) and 5 (<10%), according to the different reactivity of glycans **1a** and **2a** (Figure S1B,C).

In the case of rAg85B, the presence of eight Lys led to the formation of a different number of glycoforms, depending on the reactivity of the glycan used. The monosaccharide **1a** proved to be the most reactive and provided product **6** with seven glycoforms containing a maximum of eight glycans (Scheme 1), while the disaccharides **2a** and **3a** generated products **7** and **8** (Scheme 1) containing a maximum of six glycans (see Table S1).

Site occupancy was also defined as the procedures for carbohydrate-protein coupling that might target amino acids belonging to antigenic regions and/or affect the tertiary structure of the protein, thus influencing their immunogenic activity by perturbing the linear or conformational epitopes.

Analysis of peptides and glycopeptides obtained by chymotryptic digestion identified all the glycosylation sites at lysine residues only [21]. Recombinant TB10.4 was glycosylated at the single lysine present (Lys100), while the observed di-glycosylated product (up to 30%) was due to the secondary glycosylation of the N-terminal NH_2_-group [21]. Site occupancy in rAg85B *neo*-glycoconjugates (**6**, **7** and **8**) determined by peptide mapping (Tables S2–S4) revealed that Lys30 (corresponding to Lys23 in the native Ag85B) was the most reactive regardless of the glycan used, being glycosylated in 30–40% of the total *neo-*glycoproteins (Table 1), followed by Lys282 (corresponding to Lys275 in the native Ag85B), which was glycosylated in about 20% of the total *neo-*glycoproteins. A moderate reactivity was also observed for Lys103 and Lys123 (corresponding to Lys96 and Lys116 in the native protein, respectively), while site occupancy for the additional lysines was less than 10% (Table 1). All the investigated lyysines are located on the protein surface: the high mobility factor of N- and C-terminal regions can be partially explain the highest reactivity observed for K30 and K282.

Near- and far-UV CD spectra of the rAg85B and *neo*-glycoproteins 6 and 8 were recorded to assess folding information (see Figure S2): the results clearly show that the secondary and tertiary structure of rAg85B was not affected by the glycosylation procedure. Concerning rTB10.4 antigen, the spectra for the mannosylated derivative 4 resembled the ones for the nonglycosylated protein; however, signal intensity was lower (Figure S2).

### 2.2. Immunological Evaluation of the Neo-glycoproteins ***4**–**8***

The rTB10.4 and rAg85B proteins and their glycoderivatives were immunologically characterized on three different groups of subjects, including: (a) patients with microbiologically documented, active TB (as a group of naturally infected subjects with a high level of immunological response); (b) subjects vaccinated with *M. bovis* BCG (as a group of subjects with documented infection of anti-tuberculosis vaccine and presenting a medium or low response to the antigens under investigation); and (c) healthy, non-BCG vaccinated subjects and without any history of TB exposure as control.

**Figure 1:**
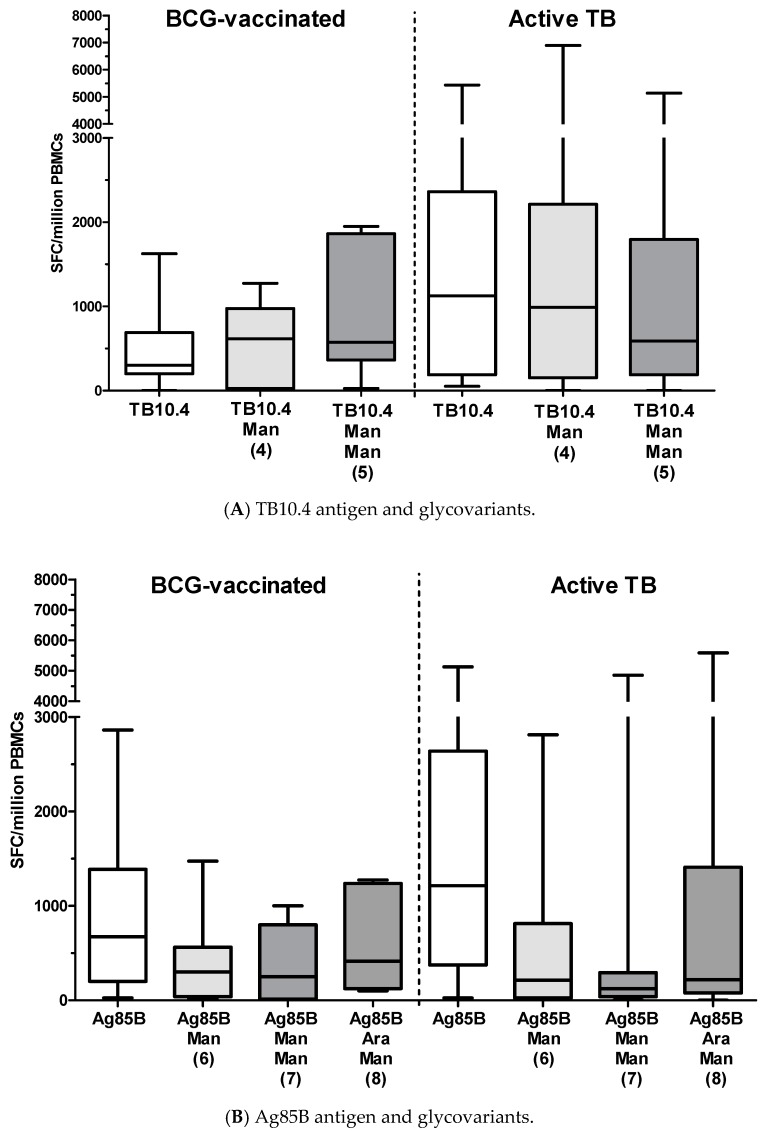
T-cell responses to: rTB10.4 (**A**); and rAg85B (**B**) antigens and glycoderivatives. Data are presented as min to max value and boxplot of 25th–75th percentile of the spot-forming cells (SFC) per million PBMCs obtained by ELISPOT in BCG-vaccinated subjects (BCG-vaccinated) and active TB patients (Active TB). rTB10.4 antigen (white box), monomannose conjugate **4** (light gray), and di-mannose conjugate **5** (medium gray). rAg85B antigen (white box), monomannose conjugate 6 (light gray), di-mannose conjugate **7** (medium gray), and arabinose-mannose conjugate **8** (dark gray).

The analysis of the T-cell response to rTB10.4 and rAg85B proteins showed that response was relevant for active TB patients and BCG vaccinated subjects (Figure 1) compared to healthy controls, as expected from previous studies [22]. Furthermore, the T-cell response to the TB10.4 was not significantly influenced by the glycosylation (Figure 1A) for both BCG-vaccinated subjects and active TB patients (Wilcoxon’s paired test, *p* > 0.05 all comparisons). In BCG-vaccinated subjects, a tendency to increase was observed for *neo-*glycoproteins **4** and **5**, although responses were not significantly higher than in the nonconjugated counterparts (*p* > 0.05 all comparisons).

In contrast, in the case of Ag85B glycosylation, the T-cell response was strongly reduced. In particular, T-cell response in active TB patients to the glycoderivatives **6** and **7** (bearing mannose and mannose-1-6-mannose) showed a marked reduction (*p* < 0.05 all comparisons), while the reduction of T-cell response to the *neo*-glycoprotein **8** (bearing arabinose-1-6-mannose) was less evident (Figure 1 panel B). A similar effect was observed in BCG-vaccinated subjects with all glycoconjugated products tested.

Analysis of the antibodies directed against rTB10.4 protein and its glycoconjugated derivatives **4** and **5** showed a general lack of response against all products tested regardless of the group of subjects considered (see Figure S3), which is in good agreement with the minor antibody reactivity reported for this MTB protein [24,25].

**Figure 2:**
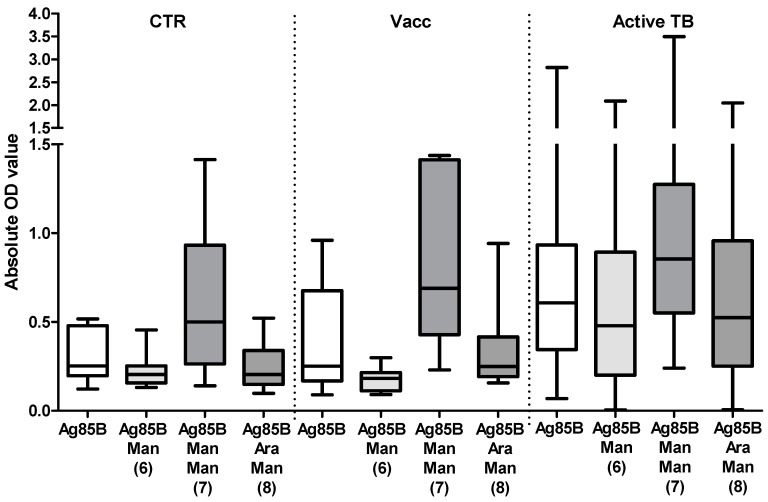
Antibody response to rAg85B and the glycoderivatives tested is presented as min to max value and boxplot of 25th–75th percentile of the absolute optical density (OD) value obtained by ELISA assay in healthy controls (CTR), BCG-vaccinated subjects (Vacc) and active TB patients (Active TB). rAg85B antigen (white box), monomannose conjugated (light gray), di-mannose conjugated (medium gray), and arabinose-mannose conjugated (dark gray).

On the other hand, rAg85B protein and its glycoconjugate derivatives showed a good antibody response in both TB and BCG-vaccinated patients (Figure 2). In the context of the observed variable response to the rAg85B antigen likely due to single patient MTB load and genetic background [26], glycosylation had a variable effect on antibody reactivity, depending on the glycan moiety introduced on the protein surface. In this context, the introduction of one mannose only (product **6**) tends to reduce partially the antibody recognition of Ag85B. Similar results were obtained when glycosylation was performed with arabinose-1-6-mannose (product **8**). This effect was not observed following glycosylation with mannose-1-6-mannose (product **7**), but, instead, a general increase in the antibody recognition was observed in most of the BCG-vaccinated and active TB subjects.

### 2.3. In Silico Prediction of T- and B-Cell Epitopes of TB10.4 and Ag85B

**Figure 3:**
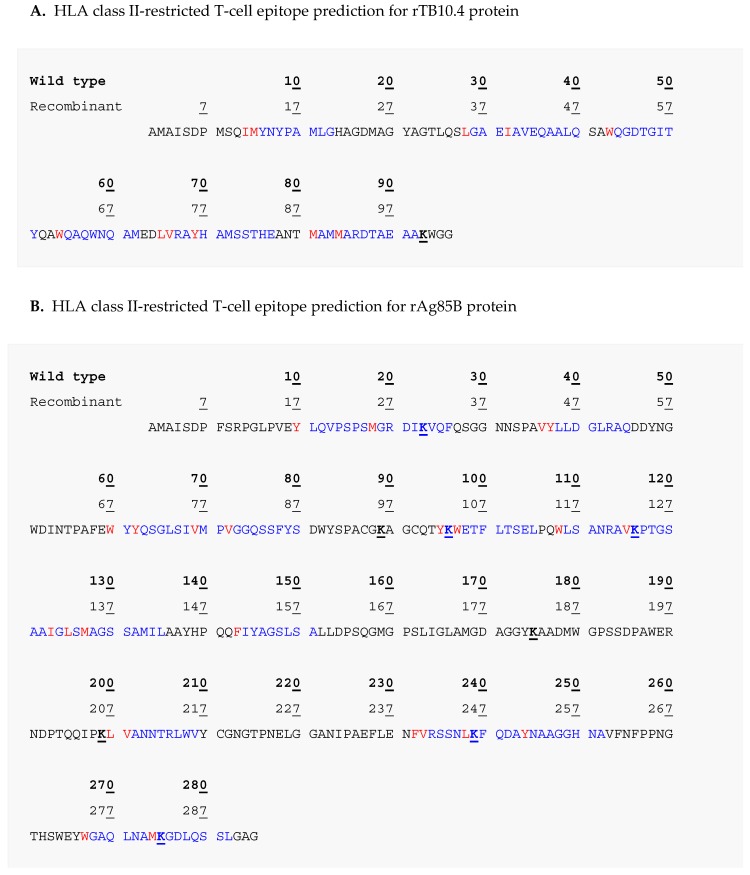
Prediction of HLA class II-restricted T-cell epitopes for the pool of frequently observed HLA alleles covering more than 90% of human populations [27] has been performed as described previously [28] by quantitatively implemented peptide-binding motif analysis at a binding capability equivalent to the top 3% of the binding peptides for each tested allele. Epitope results for all the HLA class II alleles are graphically reported on the protein sequences by indicating in blue the T-cell epitope sequence areas and in red the amino acid acting as P1 anchor of the epitope to the HLA molecule(s). Lysines are indicated in bold and underlined. Recombinant TB10.4 and Ag85B include the N-terminal tag: AMAISDP.

To evaluate the contribution of the different Lys residues in the T- and B-cell epitope formation of rTB10.4 and rAg85B antigen proteins, different in silico methods were used. Sites belonging to continuous epitopes, for T- and B-cell, or discontinuous epitopes, for B-cell, were also investigated. For this study, the primary sequence and the available three-dimensional structure of the native proteins were considered and the data correlated with the immunological activity observed with the different *neo-*glycoproteins. Figure 3 shows the predicted T-cell epitopes for the native rTB10.4 and rAg85B proteins for the set of human leucocyte antigen (HLA) class II alleles covering more than 90% of human populations [27] by quantitatively implemented peptide-binding motif analysis [28] at a binding capability equivalent to the top 3% of the binding peptides for each tested allele. In the T-cell epitope prediction of rTB10.4 protein (Figure 3A), the only target for glycosylation Lys93 (corresponding to Lys100 in the recombinant TB10.4) is not included in any T-cell epitope. For this reason, the glycosylation poorly affects the affinity in binding of the T-cell epitope M84-A92 to HLA class II alleles.

On the other hand, the T-cell epitope prediction of the rAg85B (Figure 3B) indicates that, out of the eight lysines present in the sequence of the native protein, five (Lys23, -96, -116, -239, and -275) are involved in various HLA class II promiscuous T-cell epitopes. In contrast, Lys89 and 175 are not involved in forming T-cell epitopes, while Lys199 is placed at the limit of one epitopic sequence (200–209).

Notably, the T-cell response results are also in line with the T-cell epitope prediction for Ag85B. In fact, among the five lysines directly involved in various T-cell epitopes, four (Lys30, -103, -123 and -282 considering the sequence of the recombinant protein) were glycosylated to a significant extent. In particular, position 30 proved to be the most reactive glycosylation site (up to 42% of the rAg85B was glycosylated at this site) followed by lysines at position 103, 123, and 282 (glycosylated in 10–20% of the total protein). Therefore, glycosylation of these four lysines might completely prevent recognition of T-cell epitopes of Ag85B.

For TB10.4 only the T-cell epitopes were considered for prediction studies since this MTB protein naturally showed poor antibody recognition [24,25]. The computational prediction of B-cell epitopes of the native Ag85B was instead performed based on its three-dimensional structure (pdb: 1F0N) by five different algorithms (Table S5), employing various approaches aimed to define protein surface patches with characteristics that should be recognized by antibodies.

**Figure 4:**
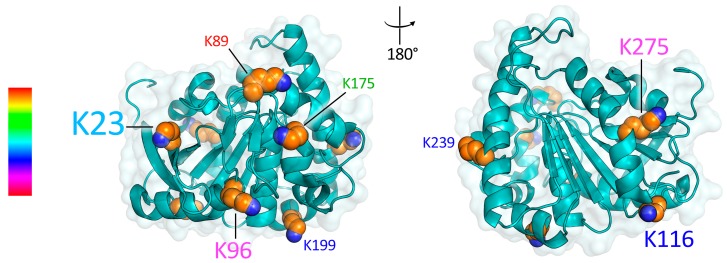
Representation of the structure of rAg85B (pdb code 1f0n) and involvement of lysines in glycosylation and in formation of B-cell epitopes. Lysine labels are marked in a different size based on the glycosylation level (large: >20%: medium: 10–20% range; small: <10%; see Table 2 for details) and by different colors based on the number of prediction methods that identified their presence as part of epitopes (red: 5; green: 4; light blue: 3; blue: 2; purple: 1; see Table 2 for details).

Table 2 summarizes the propensity of the different lysines in native Ag85B (Figure 4) to belong to B-cell epitopes. Lys89 (Lys96 in rAg85B) is predicted to be involved in B-cell epitopes by all five prediction methods. Similarly, Lys175 (Lys182 in rAg85B) is indicated by four out of five prediction systems. These two lysines belong to the same surface patch that might act as a single B-cell epitope (Table 2), but their glycosylation is targeted in less than 10% of the total proteins and, consequently, does not affect antibody recognition of the glycosylated protein.

Lys23 (Lys30 in rAg85B) is the third lysine (Figure 4), in terms of propensity of being involved in B-cell epitope formation, as predicted by three out of five methods in various protein surface patches. Other lysines appear to have a less relevant propensity to be involved in the formation of B-cell epitopes (Table 2). The abundant glycosylation of Lys23 (corresponding to Lys30 in the recombinant protein) could explain the partial reduction of the B-cell activity observed after glycosylation of rAg85B. As observed for the glycoconjugated product **7**, the glycosylation of the epitopes containing Lys30 might be compensated by additional interaction with antibodies specific for mannose (or polymannanes), induced by the high number of mannose molecules bonded on the protein surface or by the generation of *neo-*-epitopes formed by the Ag85B and the di-mannose.

## 3. Experimental Procedures

### 3.1. Synthesis of IME Mono- and Disaccharides ***1a**–**3a***

Synthesis of 1-thio-*S*-cyanomethyl mono- and disaccharides **1**–**3** and of the corresponding mono- and disaccharides **1a**–**3a** were performed according to the procedure previously reported [22]. Briefly, the peracetylated mannose bearing a thiocyanomethyl group at the C-1 position (**1**) was submitted to regioselective hydrolysis catalyzed by *Candida rugosa* lipase. The monodeprotected compound in C-6 position obtained has been then considered as intermediate for the synthesis of peracetylated arabino-mannopyranoside (**2**) and dimannopyranoside (**3**). The compounds **1**–**3** were treated with sodium methoxide. After 48 h, the reaction mixture was concentrated *in vacuum* and the solid formed was analyzed by DI-ESI-MS to evaluate the degree of conversion into IME glycosides. The yields of IME products were calculated as the ratio between the relative abundance of the activated form and the total ion intensities.

### 3.2. Preparation of MTB Proteins and Neo-glycoproteins ***4**–**8***

TB10.4 and Ag85B immunogenic proteins from *M. tuberculosis* were produced in a recombinant form in *Escherichia coli* (*E. coli*) as reported in [23] using *E. coli* BL21(DE3) cells transformed with the pET32b-Trx-TB10.4 and pET32b-Trx-Ag85B plasmids encoding for Trx-TB10.4 and Trx-Ag85B proteins, respectively. Trx-TB10.4 and Trx-Ag85B were isolated by a single chromatographic step on a nickel-affinity column (HiTrap Chelating, GE Healthcare) and maturation was performed using recombinant enterokinase (EK), followed by a further HiTrap Chelating chromatography. The removal of endotoxins was assessed by the E-TOXATE test (Sigma-Aldrich, St. Louis, MO, USA).

Purity and conformation of the recombinant protein used in this study were assessed by SDS-PAGE, DI-ESI-MS, and CD, as previously described [23,29]. Prior to glycosylation and MS analysis, buffer composition of the purified proteins was modified by ultrafiltration on Amicon^®^ Ultra centrifugal filters with a nominal molecular weight limit (NMWL) of 3 or 10 kDa.

The glycosylation reactions were conducted at 25 or 37 °C using IME-glycosides **1a**–**3a** under conditions previously optimized for product **4** [21]. The products **4**–**8** were analyzed by DI-ESI-MS and CD under the same conditions used for the nonglycosylated proteins.

### 3.3. Chymotryptic Digestion of Neo-glycoproteins ***4**–**8*** and On-Line Solid-Phase Extraction (SPE)-LC–MS Analysis

The chymotryptic digestion (specific cleavage at carboxy-terminal position of methionine, tyrosine, phenylalanine, tryptophan, and leucine residues) was carried out according to the procedure previously described for product **4** [21].

The glycopeptides obtained were selectively extracted by using an on-line method [30] optimized for the analysis of product **4** [21]. Briefly, glycopeptides were selectively on-line extracted on a Hypersyl Hypercarb trap column (10 × 4 mm I.D.; Alltech Associates) using a 10 min desorption with 80% A (ACN + 0.05% TFA)/20% B (H_2_O + 0.05% TFA) at 100 µL min^−1^, then separated on an Amide-80 column (5 µm, 80 Å, 125 × 2 mm I.D.; Tosoh Biosciences) by a gradient HPLC analysis as follows: from 30% to 57% B in 22 min with a flow rate of 100 µL min^−1^. Glycopeptides were revealed by an LTQ linear ion trap MS with an ESI source (Thermo Finnigan, SanJose, CA, USA). Mass spectra were generated in positive ion mode and MS^2^ and MS^3^ spectra were obtained by CID.

The spectra were deconvoluted using BioworksBrowser (Thermo Electron, revision 3.1) and the abundance of the different species defined by the relative intensity of the corresponding peaks in the deconvoluted spectra. The accuracy of mass determination was calculated by comparing the experimental value with the one calculated from the amino acid sequence by “Peptide mass calculator” on IonSourceMS (www.ionsource.com).

The glycosylation yields were calculated as the ratio between the relative abundance of each glycoform and the total ion intensities of the pattern in the deconvoluted spectrum.

Glycopeptides were identified using Bioworks Browser by comparing experimental data with protein FASTA sequences and considering the glycan moiety as differential modifications of the lysine residues. Only the identification with a X-corr greater than 1 were considered and, to avoid false positives, the MS^2^ and MS^3^ spectra of all species recognized as glycopeptides were manually evaluated.

### 3.4. T-Cell Epitope Prediction

HLA class II-restricted T-cell epitopes for the pool of frequently HLA alleles covering more than 90% of human populations [27] were predicted by performing quantitatively implemented peptide-binding motif analysis as previously described [28] at a binding capability equivalent to the top 3% of the binding peptides for each tested allele. Briefly, the primary protein sequences for the TB10.4 (Rv0288) and Ag85B (Rv1886c) were retrieved from the tuberculosis list database (http://tuberculist.epfl.ch/index.html). Each nonamer of the proteins was scored for binding capability to most frequent HLA class II alleles in human population by using quantitative binding matrices. Nonamers with binding score equivalent or above the top 3% of the binding peptides for the tested alleles were selected as potential epitopes.

### 3.5. B-Cell Epitope Prediction

Propensity of continuous and discontinuous B-cell epitopes for Ag85B was determined by analyzing the crystal structure of the protein (pdb 1F0N), performed with five different algorithms including: (1) SEPPA, Spatial Epitope Prediction of Protein Antigens server [31] (http://lifecenter.sgst.cn/seppa/), a tool for conformational B-cell epitope prediction which employs the three-dimensional structure of the query protein attributing to each residue a score according to its neighborhood residues; (2) EPCES, prediction of antigenic Epitopes on Protein surfaces by ConsEnsus Scoring server [32] (http://sysbio.unl.edu/EPCES/) employs six different scoring functions (residue epitope propensity, conservation score, side-chain energy score, contact number, surface planarity score, and secondary structure composition); (3) ELLIPRO, an antibody epitope prediction based on protrusion of the protein surface patches [33] (http://tools.iedb.org/ellipro/); (4) DiscoTope 2.0, a structural based B-cell epitope prediction system based on solvent accessibility and number of neighborhood contacts [34] (http://tools.iedb.org/stools/discotope/discotope.do); and (5) NeutraCorp™ (ProxAgen Ltd, Sofia, Bulgaria), a software for discontinuous B-cell epitope predictions based on structural data of protein of interest, combining protein surface patch identification as for ElliPro prediction system [33] with the identification of the amino acid contribution to the surface and their propensity in belonging to B-cell epitopes as for Discotope prediction system [34]. In addition, Neutracorp allows the tuning of the thresholds used for each parameter, for varying accuracy of prediction. For the purpose of this work thresholds were setting to allow maximum specificity and sensitivity of the prediction system to 85%.

### 3.6. Immunological Studies: Study Population

The study population included 39 subjects. Twenty-four were patients with newly diagnosed, untreated, active pulmonary TB; seven were healthy BCG-vaccinated individuals; and eight were healthy controls without any history of TB exposure. Study subjects were recruited at the Department of Infectious Diseases of the Fondazione IRCCS-Policlinico San Matteo, Pavia, Italy, after informed consent was obtained. The diagnosis of active TB was confirmed by *M. tuberculosis* culture isolation. Demographic data of the studied population are shown in the Supplementary Materials (Table S6). For all study participants, peripheral venous blood was obtained for serum samples and peripheral blood mononuclear cells (PBMC) were prepared and stored as previously described [23].

### 3.7. Immunological Studies: ELISA and ELISPOT Assays

Antibodies directed against TB10.4 and Ag85B proteins and their glycoderivatives, as well as the memory T-cell response directed against the same antigens in all the study population, were determined as previously described [23].

Data are expressed using mean and standard deviation of the mean or median and percentiles, as appropriate. Groups were compared using Mann–Whitney and χ^2^ tests. A *p* value below 0.05 was considered significant. All tests were performed using the GraphPad Prism 4.0 (Graphpad software, San Diego, CA, USA) software package.

## 4. Conclusions

Chemical glycosylation of MTB proteins (such as Ag85-antigens) with immunogenic oligosaccharides has been proposed as a new strategy for developing efficient vaccines against tuberculosis [7]. However, glycosylation of the epitopes should be avoided to take full advantage of the conjugation of immunogenic proteins and oligosaccharides. Consequently, in the design of an efficient *neo*-glycovaccine, it is mandatory to characterize the protein epitopes and investigate the effect of glycosylation on the biological activity.

For this reason, the immunogenic activity of two recombinant MTB proteins rTB10.4 and rAg85B) and new semi-synthetic *neo*-glycoproteins obtained by glycosylation with different glycans activated with IME reactive group (targeting reactive lysines) was investigated in this work, including characterization of the sequential and structural epitopes. In particular, the involvement of the different lysines of rTB10.4 and rAg85B in the formation of B- and T-cell epitopes was established by correlating the biological activity of the different glycoconjugated products with the analytical characterization of the glycosylation sites and in silico prediction of the epitopic sites.

The TB10.4 protein preferentially induces T-cell response rather than being recognized by antibodies [24,35] and the only lysine (Lys100, considering the sequence of the recombinant protein) is not involved in the epitope. Consequently, glycosylation of rTB10.4 has no impact on the immunogenic activity and this protein could be considered an optimal target for the design of glycoconjugated vaccines, although the lack of B-cell activity represents an important constraint.

In contrast, rAg85B presents both a strong antibody and cellular antigenic activity. However, glycosylation of the recombinant rAg85B strongly influences its T-cell response. For this protein, Lys30 (corresponding to Lys23 in the native protein) proved to be the most important site because it is involved in the formation of both T- and B-cell epitopes and represents the main glycosylation site (glycosylated in 30–40% of the total protein). T-cell response to this protein is strongly affected by the glycosylation because additional lysines also included in different epitopes (Lys103, -123, and -282, considering the sequence of rAg85B) can be glycosylated in 10–20% of the total proteins. In contrast, further lysines predicted as a part of various B-cell epitopes were poorly glycosylated by reaction with all IME-glycans. For this reason, antibody interaction of Ag85B was only partially reduced after coupling with mono- or disaccharides. For the *neo-*glycoprotein obtained by coupling Ag85B with mannose-1-6-mannose (product **7**), antibody recognition was even improved as compared with the *non*glycosylated counterpart, probably because of an additional interaction with antibodies specific for poly-mannanes. The role of the oligosaccharide moiety in the immunogenic activity of this *neo*-glycoproteins requires further investigation including the study of more complex glycans.

The analytical approach used in the present work can be employed for epitope characterization and to study the effect of the glycoslation of reactive amino acids in larger immunogenic proteins: anyway, the number of glycosylation sites can hamper the acquisition of useful information.

The information gained in this work regarding the involvement of the different lysines in the formation of B- and T-cell epitopes of Ag85B will be used to design new, efficient, glycoconjugated vaccine products active against TB by joining MTB proteins with antigenic oligosaccharides. Accordingly, Ag85B variants can now be rationally designed in order to avoid the glycosylation of epitopes and used to prepare *neo*-glycoprotein, preserving the natural immunogenic properties of the native antigen.

## Figures and Tables

**Table 1:** Relative abundance (%) of modified residues (site occupancy) in rAg85B subjected to glycosylation with **1a**–**3a** glycosides.

Glycoside	Site Occupancy (%)							
	K30	K96	K103	K123	K182	K206	K246	K282
Man-IME **1a**	28.2	2.7	17.8	12.8	5.5	8.1	5.3	19.6
Man(1-6)Man-IME **2a**	38.1	3.8	12.2	11.4	9.4	3.7	3.8	17.7
Ara(1-6)Man-IME **3a**	41.7	2.1	13.1	12.7	4.1	1.4	5.0	19.9

**Table 2:** Propensity of lysines in native Ag85B to belong to B-cell epitopes, as resulting from different prediction systems.

	Neutracorp™					Discotope 2.0		Ellipro			EPCES	SEPPA	
Lysine *(recombinant)*	N Surface Patches	Antigenic Propensity of the Patches	Patches with Antigenic Characteristics	Surface Cluster Sequence	Antigenic Propensity	Contact Number	Discotope Score ^1^	Protusion Score ^2^	Linear Epitope	Conformational Epitope Patch ^3^	Predicted Antigenic Epitope	Antigenic Prediction Score (0–3) ^4^	Linear Epitope Sequence
**K23** (*K30*)	10	73–91%	4	Q12 P14 P16 S17 G19 R20 D21 K23 Q93 T94 Q107 W108	87%	15	−9.9	0.5	S2-K23	1	32%	1.83	Q12-K23
				E9 Y10 Q12 P14 R20 D21 K23 Q45 D46 D47 Y48	85%								
**K89** (*K96*)	5	78–91%	3	R20 D46 Q75 S84 P85 K89 A90 C92 Q93 T94 K175	88%	12	−5.9	0.94	W82-Y95	1	91% ^5^	2.48	S84-Y95
**K96** (*K103*)	12	78–90%	4	P14 P16 S17 G19 R20 D21 D81 Q93 T94 K96 T99 S103 Q107	88%	16	−10.6	0.42	-	-	14%	1.75	-
				S17 G19 R20 D46 S84 P85 A90 C92 Q93 T94 K96	86%								
**K116** (*K123*)	14	75–86%	2	K116 I144 R206 N251 K275 G276 Q279 S280 S281 G283	86%	23	−12.2	0.58	Q107-G119	1	48%	1.71	-
				N31 N32 Q107 S110 A111 R113 A114 K116 P117 T118	85%								
**K175** (*K182*)	8	67–91%	2	R20 Q75 S84 P85 K89 A90 C92 Q93 T94 K175	88%	13	−9.6	0.77	G169-D178	1	17% ^6^	1.95	G172-D185
**K199** (*K206*)	9	75–88%	2	Q195 P198 K199 A202 N203 N204 A247 G248	88%	23	−11.6	0.58	G181-N203	1	31%	1.75	-
				Y138 N157 E189 R190 Q195 P198 K199	88%								
**K239** (*K246*)	9	68–88%	1	K239 Q241 D242 N245 A246 A247 G248	88%	20	−10.7	0.66	L238-N251	1	14%	1.75	-
**K275** (*K282*)	12	70–87%	4	K116 I144 R206 N251 N272 A273 K275 G276 Q279 S280 S281 G283 G285	85%	20	−15.9	0.45	-	-	90% ^7^	1.59	-

^1^ Score is calculated by combining the contact numbers with antigenicity propensity score of the contact amino acids. Threshold value = −7.7; ^2^ Protrusion score as portion of amino acids present in the protein surface in the patch. Default threshold value = 0.5 (>50% of the amino acid residue contributes to surface); ^3^ Number of surface patches with a radius of 6 Å containing the target residue; ^4^ Propensity to generate a conformational B-cell epitope: threshold value = 1.80; ^5^ Part of the sequential epitope C87-Q93. ^6^ Close to sequential epitope C87-Q93. ^7^ Part of the patch W60 N272 A269 A273 R275 Q279.

## Supplementary Materials

The following are available online, Figure S1: FIA-MS deconvoluted spectra of the different proteins and glycoproteins obtained by glycosylation with the different glycans. Figure S2: Analysis of conformation of native and glycosylated rTB10.4 and rAg85B by CD analyses. Figure S3: Antibody response of TB10.4 antigen and glycovariants. Table S1: Glycoform composition and abundances (%) for Ag85B conjugated with different glycosides. Table S2: Peptides obtained after digestion of the *neo-*glycoproteins derived from recombinant Ag85B-Man 6. Table S3: Peptides obtained after digestion of the *neo-*glycoproteins derived from recombinant Ag85B-Man(1–6)Man 7. Table S4: Peptides obtained after digestion of the *neo-*glycoproteins derived from recombinant Ag85B-Ara(1–6)Man 8. Table S5: Data obtained in the B-cell epitope prediction for Ag85B using different in silico prediction systems. Table S6: Demographic characteristic of the study population.
