# Supplementary material for: Glycosylation of Recombinant Antigenic Proteins from Mycobacterium tuberculosis: In Silico Prediction of Protein Epitopes and Ex Vivo Biological Evaluation of New Semi-Synthetic Glycoconjugates
Source: Molecules. 2017 Jun 29;22(7):1081. doi: 10.3390/molecules22071081 (PMC6152100; doi:10.3390/molecules22071081)
Supplement: Supplementary file 1 [file molecules-22-01081-s001.zip › molecules-202013-supplymentary-final.pdf]

molecules

MDPI

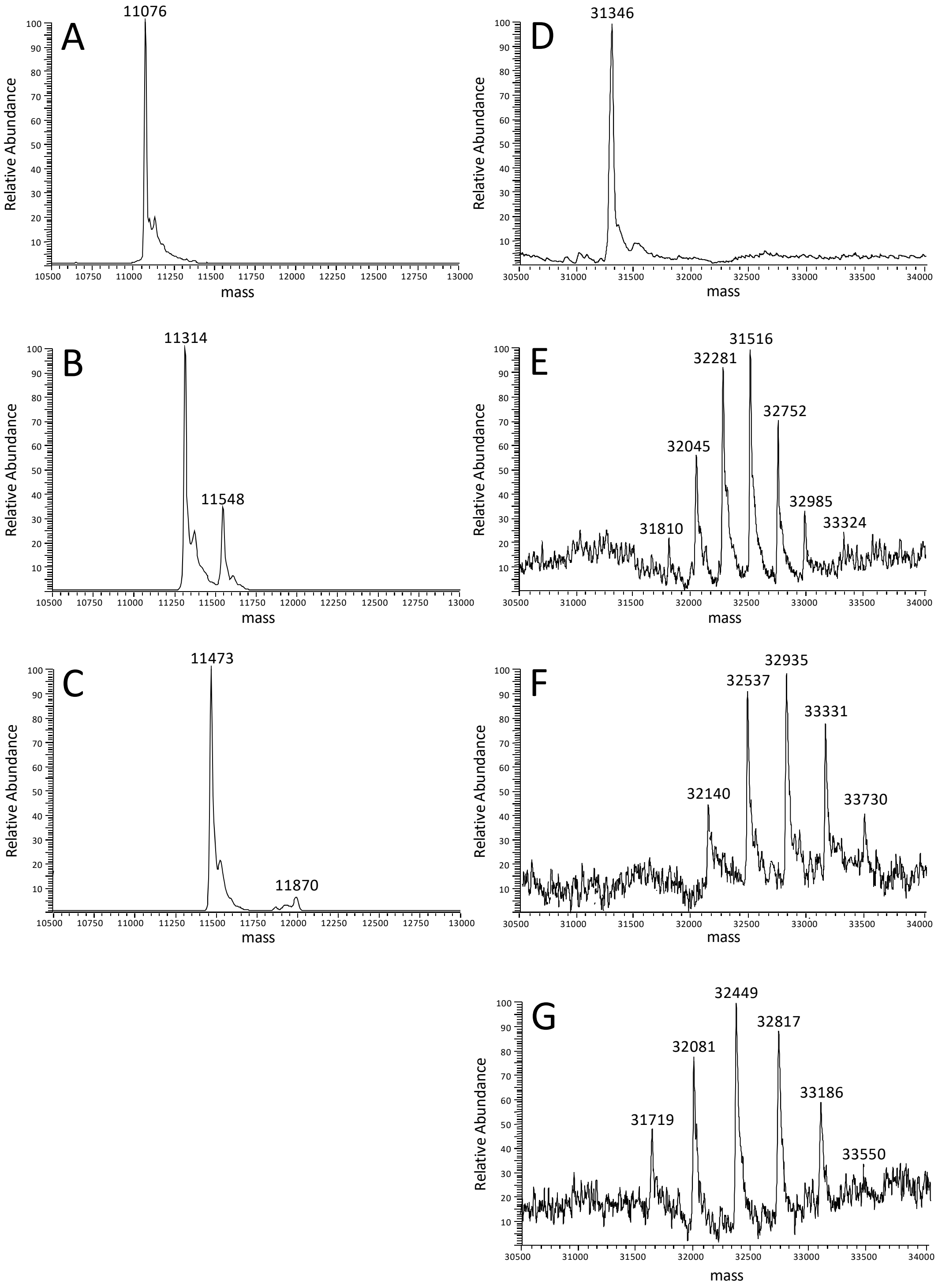

Figure S1: FIA-MS deconvoluted spectra of the different proteins and glycoproteins obtained by glycosylation with the different glycans. A) TB10.4; B) TB10.4-Man product **4**; C) TB10.4-Man(1-6)Man product **5.** D) Ag85B; E) Ag85B-Man product **6**; F) Ag85B-Man(1-6)Man product **6;** G) Ag85B-Ara(1-6)Man product **7**.

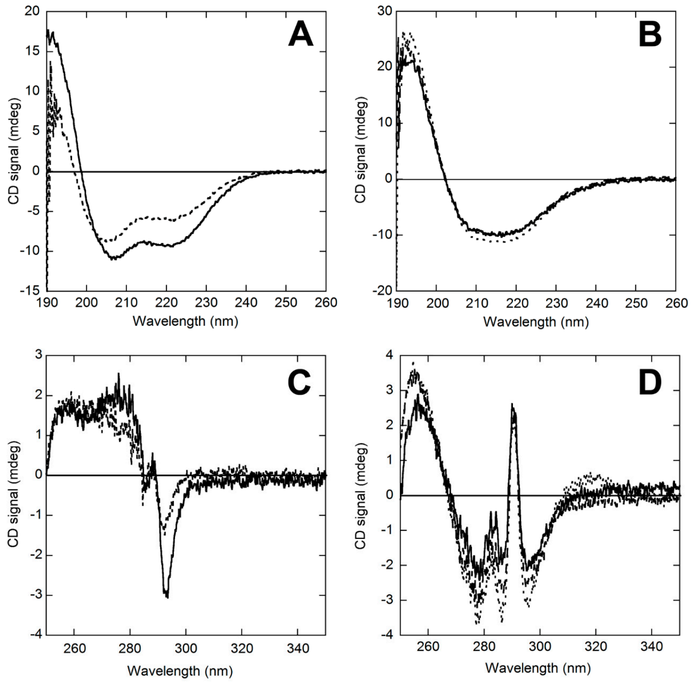

Figure S2. Analysis of conformation of native and glycosylated rTB10.4 and rAg85B by CD analyses. A) Far-UV CD spectra of rTB10.4 (continuous line) and of rTB10.4-Man (dashed line). B) Far-UV CD spectra of rAg85B (continuous line), of rAg85B-Man (dashed line) and of Ag85B-Ara-Man (dotted line). C) Near-UV CD spectra of rTB10.4 (continuous line) and of rTB10.4-Man (dashed line). D) Near-UV CD spectra of rAg85B (continuous line), of rAg85B-Man (dashed line) and of rAg85B-Ara-Man (dotted line). Protein concentrations were 0.1 mg/mL for far-UV spectra and 0.5 mg/mL for near-UV spectra. All spectra were recorded in 20 mM MOPS, 0.4 M NaCl, pH 7.0, at 15 °C.

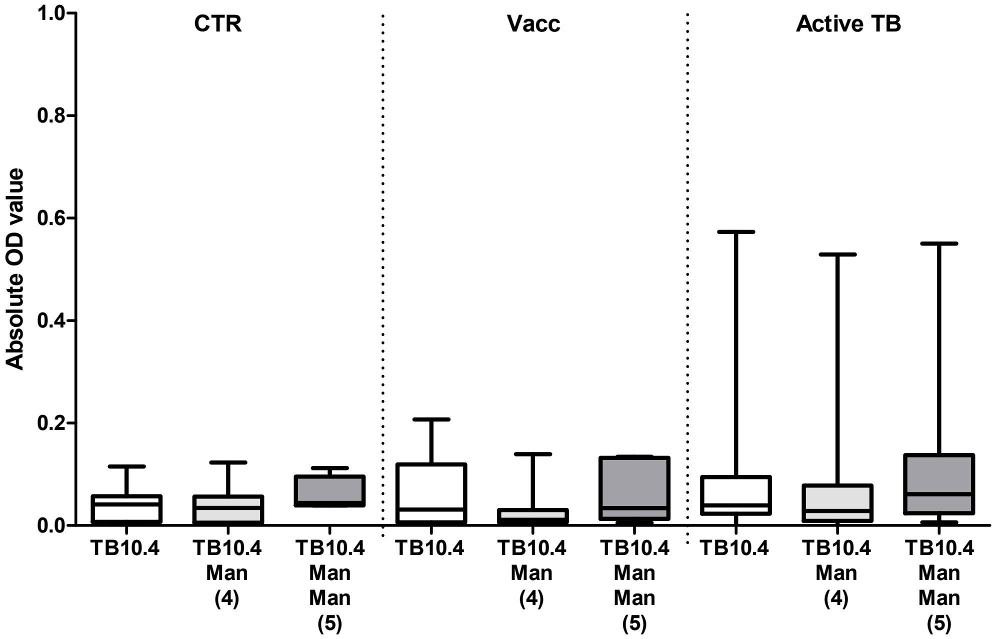

Figure S3 Antibody response of TB10.4 antigen and glycovariants

Table S1: Glycoform composition and abundances (%) for Ag85B conjugated with different glycosides.

**IME-Glycoside**	**Number of bound glycoside units[1]**								
	0	1	2	3	4	5	6	7	8
Man 1a	<1%	<1%	4.8%	12.5%	22.4%	26.6%	19.9%	9.2%	4.6%
(Exp. MW)	-	-	31810	32045	32281	31516	32752	32985	33324
Man(1–6)Man 2a	<1%	<1%	11.7%	25.1%	31.4%	21.9%	9.8%	<1%	<1%
(Exp. MW)	-	-	32140	32537	32935	33331	33730	-	-
Ara(1–6)Man 3a	<1%	6.8%	19.8%	25.4%	22.5%	19.5%	6.0%	<1%	<1%
(Exp. MW)	-	31719	32081	32449	32817	33186	33550	-	-

[1] mean value for triplicate analyses. Standard deviation < 10% in all cases

Table S2: Peptides obtained after digestion of the *neo*glycoproteins derived from recombinant Ag85B-Man **6**.

Lys	[M + H]+	[M + 2H]2+	[M + 3H]3+	Sequence	Rt (min)	Area (%)	Lys abundance (%)*
30	1329.28	665.14	443.76	S.MGRDIK*VQF.Q	14.65	5.94	28.22
	2038.08	1019.54	680.03	Y.LQVPSPSMGRDIK*VQF.Q	14.91	22.28	
96	1421.31	711.16	474.44	Y.SPACGK*AGCQTY.K	16.25	2.67	2.67
103	945.77	473.39	315.92	Y.K*WETF.L	13.71	17.77	17.77
123	1476.43	738.72	492.81	N.RAVK*PTGSAAIGL.S	15.25	8.62	12.76
	1748.69	874.85	583.56	L.SANRAVK*PTGSAAIGL.S	15.85	4.14	
182	956.82	478.91	319.61	Y.K*AADMW.G	13.89	5.46	5.46
206	2359.22	1180.11	787.07	W.GPSSDPAWERNDPTQQIPK*.L	16.84	0.12	8.14
	2756.69	1378.85	919.56	W.GPSSDPAWERNDPTQQIPK*LVAN.N	16.56	2.43	
	2870.79	1435.90	957.60	W.GPSSDPAWERNDPTQQIPK*LVANN.T	16.99	2.11	
	2971.89	1486.45	991.30	W.GPSSDPAWERNDPTQQIPK*LVANNT.R	16.99	1.90	
	3241.24	1621.12	1081.08	W.GPSSDPAWERNDPTQQIPK*LVANNTRL.W	17.57	1.58	
246	1006.81	503.91	336.27	L.K*FQDAY.N	14.80	5.34	5.34
282	1268.09	634.55	423.36	M.K*GDLQSSLGAG.-	15.74	4.48	19.64
	1399.28	700.14	467.09	L.NAMK*GDLQSSL.G	15.28	3.19	
	1470.36	735.68	490.79	N.AMK*GDLQSSLGAG.-	15.39	3.33	
	1584.46	792.73	528.82	L.NAMK*GDLQSSLGAG.-	15.74	8.65	

* The Lys abundance for each residue is calculated from the sum of percentage area of all peptides containing the same lysine.

Table S3: Peptides obtained after digestion of the *neo*glycoproteins derived from recombinant Ag85B-Man(1–6)Man **7**.

**Lys**	**[M+H]⁺**	**[M+2H]²⁺**	**[M+3H]³⁺**	**Sequence**	**Rt (min)**	**Area (%)**	**Lys abundance (%)***
**30**	2201.12	1100.62	734.24	Y.LQVPSPSMGRDIK*VQF.Q	15.71	33.00	38.19
	1492.31	746.04	497.79	S.MGRDIK*VQF.Q	15.56	5.19	
**96**	1581.16	790.98	528.33	Y.SPACGK*AGCQTY.K	17.32	3.74	3.74
**103**	1107.21	554.38	370.00	Y.K*WETF.L	15.22	11.66	11.66
**123**	1911.73	955.62	637.74	L.SANRAVK*PTGSAAIGL.S	16.66	5.66	11.01
	1639.47	819.62	546.93	N.RAVK*PTGSAAIGL.S	16.12	5.35	
**182**	1119.17	559.89	373.33	Y.K*AADMW.G	15.43	9.16	9.16
**206**	3134.94	1567.13	1045.15	W.GPSSDPAWERNDPTQQIPK*LVANNT.R	17.68	0.84	3.28
	3033.83	1516.26	1011.60	W.GPSSDPAWERNDPTQQIPK*LVANN.T	17.72	1.25	
	2919.73	1459.73	973.89	W.GPSSDPAWERNDPTQQIPK*LVAN.N	17.32	1.09	
	3404.28	1702.64	1135.74	W.GPSSDPAWERNDPTQQIPK*LVANNTRL.W	18.26	0.10	
**246**	1168.31	584.69	391.10	L.K*FQDAY.N	15.96	4.78	4.78
**282**	1633.34	817.06	545.13	N.AMK*GDLQSSLGAG.-	16.39	2.56	18.18
	1746.32	873.86	583.17	L.NAMK*GDLQSSLGAG.-	16.70	10.46	
	1431.12	715.49	477.71	M.K*GDLQSSLGAG.-	16.73	2.92	
	1561.00	781.00	521.00	L.NAMK*GDLQSSL.G	16.25	1.41	
	1762.00	881.50	588.00	Oxidised L.NAMK*GDLQSSLGAG.-	17.39	0.82	

* The Lys abundance for each residue is calculated from the sum of percentage area of all peptides containing the same lysine

Table S4: Peptides obtained after digestion of the *neo*glycoproteins derived from recombinant Ag85B-Ara(1–6)Man **8**.

Lys	[M+H]+	[M+2H]2+	[M+3H]3+	Sequence	Rt (min)	Area (%)	Lys abundance (%)*
**30**	2170.08	1085.54	724.03	Y.LQVPSPSMGRDIK*VQF.Q	15.36	35.86	41.75
	2192.99	1096.99	731.66	Y.LQVPSPSMGRDIK*VQF.Q	15.32	5.88	
**96**	1553.31	777.16	518.44	Y.SPACGK*AGCQTY.K	16.95	2.07	2.07
**103**	1077.77	539.39	359.92	Y.K*WETF.L	14.73	13.13	13.13
**123**	1608.43	804.72	536.81	N.RAVK*PTGSAAIGL.S	15.77	4.60	12.70
	1880.69	940.85	627.56	L.SANRAVK*PTGSAAIGL.S	16.34	8.10	
**182**	1088.82	544.91	363.61	Y.K*AADMW.G	14.98	4.10	4.10
**206**	2888.69	1444.85	963.56	W.GPSSDPAWERNDPTQQIPK*LVAN.N	17.00	0.44	1.36
	3002.79	1501.90	1001.60	W.GPSSDPAWERNDPTQQIPK*LVANN.T	17.42	0.38	
	3103.89	1552.45	1035.30	W.GPSSDPAWERNDPTQQIPK*LVANNT.R	17.42	0.54	
**246**	1138.81	569.91	380.27	L.K*FQDAY.N	15.47	4.98	4.98
**282**	1400.09	700.55	467.36	M.K*GDLQSSLGAG.-	16.34	1.99	19.91
	1531.28	766.14	511.09	L.NAMK*GDLQSSL.G	15.87	0.89	
	1602.36	801.68	534.79	N.AMK*GDLQSSLGAG.-	16.00	3.40	
	1716.46	858.73	572.82	L.NAMK*GDLQSSLGAG.-	16.34	12.80	
	1732.46	866.73	578.15	L. NAMK*GDLQSSLGAG.-	17.07	0.83	

* The Lys abundance for each residue is calculated from the sum of percentage area of all peptides containing the same lysine

Table S5 Data obtained in the B-cell epitope prediction for Ag85B using different *in silico* prediction systems.

See file.

Table S6. Demographic characteristic of the study population.

**Study group**	**N**	**Ethnicity**	**Country of origin**	**Immigrant**	**Age***	**Sex (M/F)**	**AFB stain# (% positive)**	**Culture$ (% positive)**
**Healthy Control**	8	All Caucasian	8 Italy	0%	26 (25-28)	1/7	Not applicable	Not applicable
**BCG vaccinated**	7	All Caucasian	6 Italy<br>1 Argentina	0%	40 (35-53)	1/6	Not applicable	Not applicable
**Active TB**	24	21 Caucasians<br><br>1 Asian<br><br>2 South Americans	9 Italy<br>7 Romania<br>3 Albania<br>1 Bulgaria<br>1 Ecuador<br>1 Moldavia<br>1 Pakistan<br>1 Philippines	62.5%	41 (29-55)	16/8	79%	100%

*Data is expressed as median and 25th-75th interquartile range.

#AFB: Acid Fast Bacilli stain

$Culture: *Mycobacterium tuberculosis* culture positive confirmation including molecular assay
